# AGING IN A GOOD WAY THROUGH THE EYES OF ALASKA NATIVE ELDERS: RESULTS FROM A 16-YEAR CBPR STUDY

**DOI:** 10.1093/geroni/igae098.1124

**Published:** 2024-12-31

**Authors:** Jordan Lewis, Lena Thompson, Steffi Kim

**Affiliations:** Chair; Co-Chair; Discussant

## Abstract

Predominantly Western-based biomedical models of successful aging have been used to research, understand, and explain successful aging among diverse populations. The presenters on this symposium have been exploring successful aging from an Alaska Native perspective or what it means to reach “Eldership,” or age in a good way, in urban and rural communities across the State of Alaska. This symposium will present findings from a 16-year study on Alaska Native successful aging. To learn about aging well among Alaska Natives, we have conducted 162 interviews with community-nominated Elders representing 21 Alaska Native communities in five regions of Alaska. Our team asked Alaska Native Elders what it means to them to age in a good way, what supports them to age where and how they want, and what they want younger people to know about aging in a good way. Dr. Jordan P. Lewis will provide an introductory overview of the 16-year study and share the element of emotional health and generativity. Dr. Steffi Kim will discuss the role of community engagement in successful aging for rural and urban Alaska Native Elders followed by Dr. Lena Thompson discussing the role of spirituality and Native ways of life as supporting their ability to meaningfully age. Dr. Maja Pedersen will discuss the role of physical health, including Indigenous approaches to staying active and aging in a good way. The symposium will conclude with a discussion on the interrelated nature of these themes and how attendees can apply these lessons to their own lives.

